# Prä‑, Pro- und Postbiotika – eine Einführung

**DOI:** 10.1007/s00106-025-01657-6

**Published:** 2025-08-05

**Authors:** Maria do Carmo Greier, Benedikt Gabriel Hofauer

**Affiliations:** Universitätsklinik für Hals‑, Nasen- und Ohrenheilkunde, Medizinische Universität Innsbruck, Anichstraße 35, 6020 Innsbruck, Österreich

**Keywords:** Orales Mikrobiom, Bioaktive Metaboliten, Dysbiose, Mundgesundheit, Mund-Darm-Achse, Oral microbiome, Bioactive metabolites, Dysbiosis, Oral health, Oral-gut axis

## Abstract

Das menschliche Mikrobiom besteht aus einer Vielzahl von Mikroorganismen, die essenzielle Funktionen für die Gesundheit erfüllen. Besonders das orale Mikrobiom spielt eine zentrale Rolle in der Mundgesundheit, indem es die Kolonisation pathogener Bakterien verhindert und das Gleichgewicht des pH-Werts aufrechterhält. Eine Dysbiose im oralen Mikrobiom wird mit der Entstehung von Karies, Parodontalerkrankungen und oralen Plattenepithelkarzinomen in Verbindung gebracht. Darüber hinaus besteht eine bidirektionale Wechselwirkung zwischen dem oralen und dem intestinalen Mikrobiom, die über die sog. Mund-Darm-Achse vermittelt wird. Zur Modulation des Mikrobioms wurden in den letzten Jahren Präbiotika, Probiotika und insbesondere Postbiotika intensiv untersucht. Postbiotika stellen eine vielversprechende Alternative dar, da sie keine lebensfähigen Mikroorganismen, sondern bioaktive Metaboliten, Zellwandfragmente oder Enzyme enthalten, die immunmodulatorische, entzündungshemmende und antimikrobielle Effekte entfalten. Insbesondere im Bereich der oralen Gesundheit zeigen Postbiotika potenzielle Vorteile, indem sie das Wachstum pathogener Keime hemmen, die Immunantwort regulieren und entzündliche Prozesse reduzieren. Aktuelle Forschungsergebnisse legen nahe, dass Postbiotika die Mundgesundheit nachhaltig verbessern. Zudem konnte in ersten Studien eine mögliche Anwendung in der unterstützenden Therapie oraler Krebserkrankungen durch ihre antitumoralen Eigenschaften gezeigt werden.

## Abstract (en)

The human microbiome consists of a diverse array of microorganisms that perform essential functions for health. In particular, the oral microbiome plays a crucial role in maintaining oral health by preventing the colonization of pathogenic bacteria and regulating pH balance. Dysbiosis of the oral microbiome has been linked to the development of caries, periodontal diseases and oral squamous cell carcinoma. Moreover, a bidirectional interaction between the oral and intestinal microbiomes is mediated through the so-called oral-gut axis. In recent years, prebiotics, probiotics and especially postbiotics have been extensively studied for their potential in microbiome modulation. Postbiotics represent a promising alternative as they do not contain viable microorganisms but bioactive metabolites, cell wall fragments or enzymes that exert immunomodulatory, anti-inflammatory and antimicrobial effects. Particularly in the context of oral health, postbiotics show potential benefits by inhibiting the growth of pathogenic microbes, regulating immune responses and reducing inflammatory processes. Recent research results suggest that postbiotics can sustainably improve oral health. Furthermore, preliminary studies indicate their potential application as an adjunct therapy for oral cancer due to their antitumor properties.

Das menschliche Mikrobiom besteht aus einer Vielzahl von Mikroorganismen. Jeder Bakterienstamm hat ein Genom mit Tausenden von Genen, welches eine größere genetische Vielfalt und damit Flexibilität bietet als das menschliche Genom. Heute weiß man, dass Veränderungen im Mikrobiom und im mikrobiellen Metabolom sowie deren Interaktion mit dem Immun‑, Hormon- und Nervensystem mit einer Vielzahl von Krankheiten korreliert sind. Daher spielt das Mikrobiom eine entscheidende Rolle für die Gesundheit.

## Das Mikrobiom

Im Gegensatz zur Mikrobiota, also der Gesamtheit aller Mikroorganismen in einer bestimmten Region des menschlichen Körpers, bezieht sich das Mikrobiom auch auf deren Gene, Stoffwechselprodukte und die komplexen Interaktionen innerhalb der mikrobiellen Umgebung. Ein Ungleichgewicht der Mikroorganismen, bekannt als Dysbiose, wird mit verschiedenen Erkrankungen wie Magen-Darm-Störungen, Stoffwechselerkrankungen und Immunsystemstörungen in Verbindung gebracht [16, 17]. Neue Forschungsergebnisse zeigen das Potenzial mikrobiombasierter Therapien zur Wiederherstellung des mikrobiellen Gleichgewichts. Hierfür kommen besonders häufig Synbiotika zum Einsatz. Diese sind eine Kombination aus Probiotika (lebendende Bakterien) und Präbiotika (unverdauliche Nahrungsbestandteile), die synergistisch wirken, um ein stabiles und gesundheitsförderndes Mikrobiom zu unterstützen [32].

**Abb. 1:**
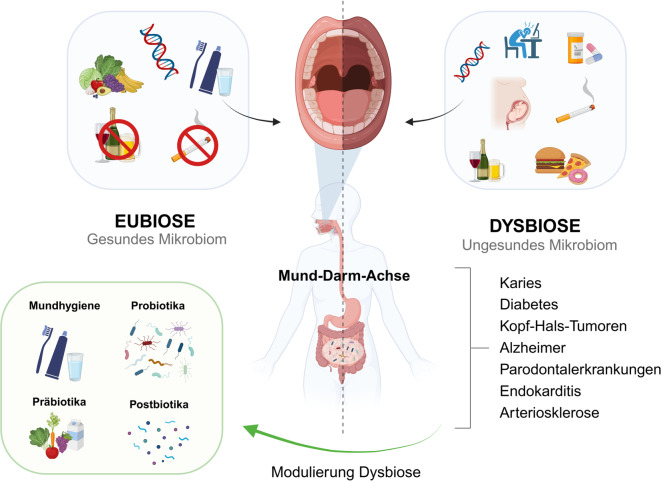
Eubiose und Dysbiose des oralen Mikrobioms und des Darmmikrobioms: Verschiedene Faktoren beeinflussen das orale Mikrobiom, welches wiederum das Darmmikrobiom über die Mund-Darm Achse beeinflussen kann. Eine Dysbiose kann verschiedene Erkrankungen begünstigen. Dies kann jedoch moduliert werden, um ein mikrobielles Gleichgewicht wiederherzustellen. (Erstellt mit BioRender.com)

Die Entwicklung des menschlichen Mikrobioms beginnt bereits mit der Geburt, da Säuglinge durch vaginale Entbindung oder Kaiserschnitt Mikroben von ihrer Mutter erhalten. Vaginal entbundene Säuglinge erwerben ein Mikrobiom, das reich an *Lactobacillus*-Spezies ist, während per Kaiserschnitt geborene Säuglinge anfangs eine höhere Anzahl hautassoziierter Mikroben wie *Staphylococcus* aufweisen. Dies wiederum kann sich auf das Immunsystem auswirken [24]. Mit der Einführung fester Nahrung verändert sich die Zusammensetzung des Mikrobioms, welche eine größere Vielfalt an Bakterien umfasst, sodass es bereits im frühen Kindesalter dem Darmmikrobiom eines Erwachsenen ähnelt. Das humane Mikrobiom entwickelt sich jedoch im Laufe des Lebens kontinuierlich weiter, da Faktoren wie Ernährung, Lebensstil, Umweltfaktoren und Medikamente einen signifikanten Einfluss auf die Zusammensetzung und Funktion des Mikrobioms ausüben (Abb. 1; [1]).

Im menschlichen Körper weist die Darmmikrobiota im Vergleich zu anderen Bereichen des Körpers die höchste Anzahl und größte Vielfalt an Bakterien auf. Jedoch beeinflusst nicht nur das Darmmikrobiom die Gesundheit, sondern auch das orale Mikrobiom, das Mikrobiom der Haut und des Genitalbereichs. V. a. das orale Mikrobiom, welches eine vielfältige und dynamische bakterielle Gemeinschaft aufweist, ist besonders bei der Aufrechterhaltung der Mundgesundheit wichtig, indem es die Besiedlung schädlicher Bakterien hemmt und zur Aufrechterhaltung des pH-Gleichgewichts beiträgt [37]. Eine Dysbiose des oralen Mikrobioms kann zu Karies und/oder Parodontalerkrankungen führen, was wiederum längerfristig wesentlich zur Entstehung von oralen Plattenepithelkarzinomen beitragen kann [6]. Darüber hinaus hat die Verbindung zwischen dem oralen und dem Darmmikrobiom, die sog. Mund-Darm-Achse, in den vergangenen Jahren an Bedeutung gewonnen, da Veränderungen im oralen Mikrobiom das Darmmikrobiom beeinflussen [23]. In weiteren Studien konnte gezeigt werden, dass Parodontalpathogene im oralen Mikrobiom das Schlaganfallrisiko erhöhen können, indem sie Erkrankungen wie Fettleibigkeit, gestörte Blutfettwerte, Arterienverkalkung, Bluthochdruck, Alzheimer und Diabetes verschlimmern bzw. begünstigen können [26, 50]. Auch Entzündungen wie Parodontitis wurden mit Erkrankungen wie Diabetes und Alzheimer in Verbindung gebracht ([36]; Abb. 1).

## Präbiotika

Präbiotika werden als unverdauliche Nahrungsbestandteile beschrieben, welche gezielt von Darmbakterien abgebaut bzw. fermentiert werden. Daher können Präbiotika zu spezifischen Veränderungen in der Zusammensetzung und/oder Aktivität der gastrointestinalen Mikrobiota führen und dadurch Vorteile für die Gesundheit des Wirts mit sich bringen [4].

In der Literatur wurden bisher mehrere Definitionen von Präbiotika verwendet, jedoch gibt es insgesamt keine allgemeingültige Beschreibung. Daher wurden Kriterien genannt, welche für die Einstufung einer Verbindung als Präbiotikum erforderlich sind [13]. Präbiotika müssen den sauren pH-Wert des Magens überstehen, dürfen nicht durch Verdauungsenzyme abgebaut oder im Darm aufgenommen werden, sollten von der Darmflora fermentiert werden und gezielt das Wachstum bestimmter Darmbakterien fördern. Zu den Präbiotika zählen Verbindungen aus der Gruppe der Kohlenhydrate wie resistente Stärke, Galaktooligosaccharide, kurz- und langkettige Fruktane (Oligosaccharide und Inulin) und Laktulose. Gute Quellen dafür sind v. a. Lebensmittel wie Knoblauch, Zwiebeln, Hafer, Weizen, Hülsenfrüchte, Äpfel und Sojabohnen [9].

Für die Entwicklung des Darmmikrobioms sind Präbiotika von großer Bedeutung. Diese Entwicklung beginnt bereits bei Neugeborenen und hängt stark von der Art der Ernährung ab. Über die Muttermilch werden die ersten natürlichen Präbiotika, die sog. humanen Milcholigosaccharide (HMO), weitergegeben. Daher weisen Säuglinge, die gestillt werden, im Gegensatz zu Säuglingen, die Säuglingsanfangsnahrung erhalten, ein Mikrobiom auf, welches von Bifidobakterien und Laktobazillen dominiert wird [8, 49]. HMO beeinflussen nicht nur das Mikrobiom im Darm positiv, sondern modulieren auch das orale Mikrobiom bei Säuglingen [2]. Präbiotika sind auch in der Lage, die Mundgesundheit durch die gezielte Förderung nützlicher oraler Mikroben aufrechtzuerhalten, indem sie als Nährstoffe für diese dienen und gleichzeitig das Wachstum pathogener Spezies hemmen, die mit Erkrankungen in Zusammenhang stehen [52]. V. a. Nitrat, das in Gemüse vorkommt, sowie Polyphenole, welche man besonders in Beeren sowie in grünem und schwarzem Tee findet, zeigten wachstums- und entzündungshemmende sowie antimikrobielle Eigenschaften gegen Karies- und Parodontitiserreger [12]. Auch Inulin oder Fruktooligosaccharide fördern das Wachstum gesundheitsfördernder Mikroorganismen im Mundraum und können dadurch die orale Gesundheit unterstützen [34]. Trotz positiver Eigenschaften können Präbiotika jedoch auch zu gastrointestinalen Nebenwirkungen wie Blähungen oder Durchfall führen, insbesondere bei hoher Dosierung oder empfindlicher Darmflora [42].

## Probiotika

Die International Scientific Association for Probiotics and Prebiotics (ISAPP) definierte den Begriff „Probiotikum“ wie folgt: „Probiotika sind lebende Mikroorganismen, die, wenn sie in angemessenen Mengen verabreicht werden, dem Wirt einen gesundheitlichen Nutzen bringen“ [13]. Ein Produkt kann deshalb nur dann als Probiotikum bezeichnet werden, wenn es lebende Mikroorganismen enthält, die entweder auf Stamm- oder auf Gruppenebene wissenschaftlich nachgewiesene gesundheitliche Vorteile bieten. Probiotika können sich in ihrer Verabreichungsform, ihrem Zielwirt, ihrem Wirkort und ihren Wirksamkeitsendpunkten unterscheiden.

Probiotika können die Gesundheit fördern, indem sie die Darmmikrobiota modulieren, die Immunreaktionen verbessern und pathogene Infektionen verhindern [13, 40]. Die wichtigsten und am häufigsten verwendeten probiotischen Bakterien stammen aus den Gattungen *Lactobacillus* und *Bifidobacterium* und tragen wesentlich zur Darmhomöostase sowie zu einer verbesserten Verdauung und Unterstützung des Stoffwechsels bei [35]. Neben der Darmgesundheit werden Probiotika auch mit positiven Auswirkungen auf das Immunsystem sowie auf die psychische, die Haut- und die Mundgesundheit in Verbindung gebracht [18]. Probiotika können Entzündungen verringern und sind in der Lage, das orale Mikrobiom zu modulieren, indem sie die Präsenz nützlicher Bakterien wie *Lactobacillus reuteri* und *Lactobacillus rhamnosus* erhöhen und gleichzeitig pathogene und kariogene Bakterien wie *Streptococcus mutans* und *Candida albicans* verringern [3, 18]. In einigen Studien der letzten Jahre konnte gezeigt werden, dass Erkrankungen wie Karies oder Zahnfleischentzündungen durch orale Verabreichung von Probiotika vermindert werden können. Dafür verwendet man beispielsweise dünne Polymerfilme (orodispersible Filme), die mit Probiotika angereichert werden und sich in der Mundhöhle auflösen oder zerfallen [5, 11]. Des Weiteren können Probiotika wie *Streptococcus salivarius K12* [10, 41] oder *Lactobacillus rhamnosus *[27, 29] im HNO(Hals-Nasen-Ohren)-Bereich zur Prävention und Reduktion rezidivierender Infektionen, wie Tonsillitis oder Otitis media, eingesetzt werden. Sie wirken durch Kolonisierung der Schleimhäute und Hemmung pathogener Keime [18].

Durch die immunmodulatorische Wirkung haben sich Probiotika in den letzten Jahren auch als geeignet für die Prävention und Behandlung von oralen Karzinomen erwiesen, da sie bestimmte Stämme pathogener Bakterien, die an der Karzinogenese beteiligt sind, hemmen und nützliche Kommensalen fördern, welche Immunwege regulieren und das Fortschreiten des Tumors beeinflussen [22]. Darüber hinaus können Probiotika und deren Stoffwechselprodukte die Apoptose in bösartigen Zellen auslösen und chronische Entzündungen lindern [25]. Um die Krebstherapie zu unterstützen und präventive Maßnahmen durch Probiotika zu erzielen, können verschiedene orale Darreichungsformen wie Kautabletten, mukoadhäsive Tabletten und Filme, Gele, Mundspülungen, orale Tropfen und Sprays verwendet werden [48, 51].

Trotz der vielen positiven Eigenschaften von Probiotika sind auch Bedenken hinsichtlich deren Verwendung aufgekommen. Insbesondere stellen die industrielle Verarbeitung und Lagerung technologische Herausforderungen dar, da sie die Zellviabilität beeinträchtigen können. Darüber hinaus ist bei Verwendung von lebenden Mikroorganismen, gerade bei gefährdeten Gruppen wie älteren und immungeschwächten Personen, Vorsicht geboten. Zudem besteht auch die Möglichkeit einer unerwünschten Beeinflussung des lokalen Mikrobioms [28]. Als Alternative dazu fokussierte man sich in den letzten Jahren häufig auf Postbiotika [31].

## Postbiotika

**Abb. 2:**
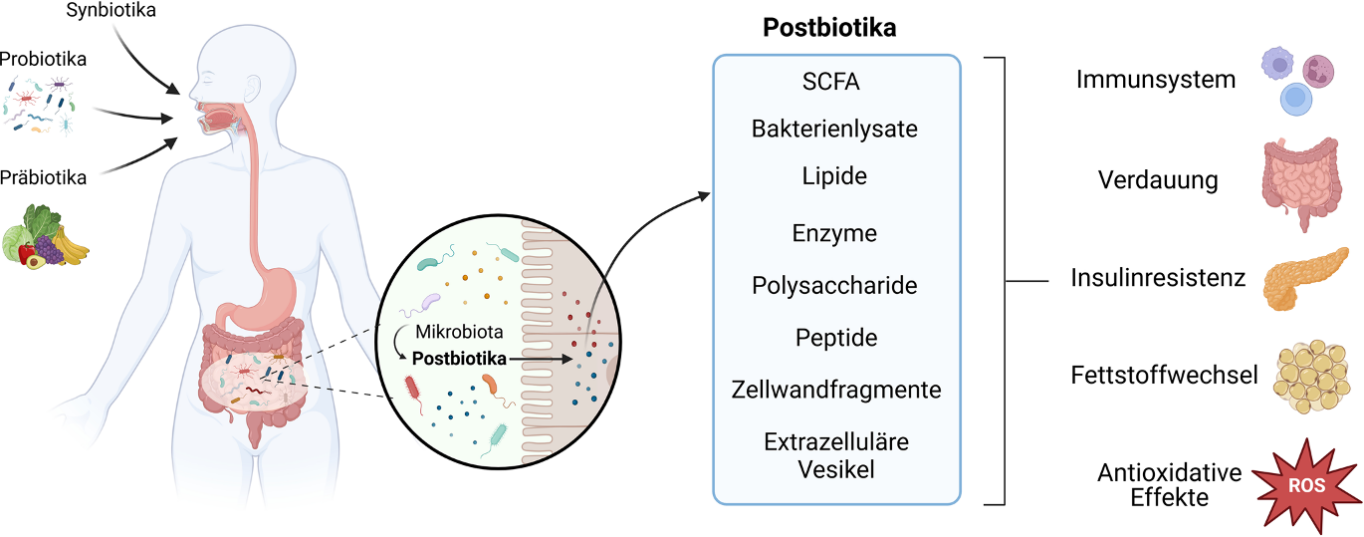
Entstehung von Postbiotika: Postbiotika werden durch Mikrobiota produziert und können über die Darmschleimhaut aufgenommen werden. Postbiotika sind in der Lage, verschiedene Erkrankungen zu beeinflussen, da sie auf das Immunsystem, den Fettstoffwechsel, die Verdauung und die Insulinsensitivität wirken. Darüber hinaus weisen Postbiotika auch antioxidative Effekte auf. *SCFA* „short-chain fatty acids“, *ROS* „reactive oxygen species“. (Erstellt mit BioRender.com)

Unter Postbiotika versteht man laut ISAPP Präparate aus unbelebten Mikroorganismen und/oder ihren Bestandteilen, welche dem Wirt gesundheitlichen Nutzen verschaffen [39]. Postbiotika sind bioaktive Verbindungen, die aus bzw. von Mikroorganismen nach deren Absterben produziert werden, von ihnen stammen oder während der mikrobiellen Fermentation eines Substrats entstehen. Der Begriff umfasst Metaboliten, Zellen sowie strukturelle Fragmente [31]. Dazu zählen v. a. Bakterienlysate, Peptide, Zellwandfragmente, Lipide und kurzkettige Fettsäuren („short-chain fatty acids“ [SCFA]; Abb. 2).

Postbiotika bieten vielfältige gesundheitliche Vorteile, darunter die Modulation der Immunantwort und die Veränderung der Zusammensetzung der Darmmikrobiota. Darüber hinaus beeinflussen sie die Zytokinproduktion, fördern die Sekretion antimikrobieller Peptide und regen die Muzinproduktion an, was die Integrität der Darmbarriere unterstützt [46]. Schließlich können sie auch Stoffwechselparameter wie Insulinsensitivität und Cholesterinspiegel verbessern und antioxidativ wirken [15]. Postbiotika gelten aufgrund ihrer guten Säure-Basen- und Hitzestabilität, ihrer einfachen Lagerung und Verwendung, ihrer hohen Sicherheit sowie ihrer kürzeren Passage in der Mundhöhle besser bzw. effizienter als Probiotika. Sie können daher gängigen Produkten wie Zahnpasta, Kaugummi oder Lutschtabletten zugesetzt werden, wodurch sie wiederum für neue Therapieformen, besonders im Hinblick auf orale Erkrankungen, von Interesse sind [30].

Postbiotika sind in der Lage, orale Pathogene wie *Streptococcus mutans* zu hemmen, indem sie den pH-Wert senken und die Produktion von Bakteriozinen fördern. Diese können gezielt das Wachstum pathogener Keime hemmen und fördern so ein stabiles orales Mikrobiom. Hierzu zählen beispielweise Bakteriozine wie Salivaricin A2 und Salivaricin B aus *Streptococcus salivarius K12*, welche zur Prävention rezidivierender Pharyngitis verwendet werden [44]. Zudem können Postbiotika die Produktion von Immunglobulin A (IgA) im Speichel anregen, was die orale Immunität stärkt. Ihre entzündungshemmenden Eigenschaften können zudem bei Parodontalerkrankungen helfen, indem sie Entzündungen reduzieren und die Heilung fördern [14, 38]. Darüber hinaus konnten in In-vitro-Studien vielversprechende Ergebnisse für Postbiotika hinsichtlich der antimikrobiellen Wirkung, der Verhinderung der Biofilmbildung und der Verringerung der Transkription von virulenzassoziierten Genen einiger Parodontopathogene gezeigt werden [7, 19]. Daher können Postbiotika bei der Prävention und Behandlung verschiedener Erkrankungen wie Karies, Halitosis, Parodontitis und Gingivitis mittels Mundspülungen, Zahnpasten, Kaugummis, Lutschtabletten und Sprays zum Einsatz kommen [5, 47].

Postbiotika wurden auch mit antitumoralen Eigenschaften in Verbindung gebracht. V. a. Postbiotika aus *Lactobacillus acidophilus *und *Bifidobacterium longum* zeigten eine signifikante Antitumoraktivität, indem sie die zelluläre Immunität modulieren. Einige Postbiotika sind auch in der Lage, die Wirksamkeit und Zytotoxizität von Chemotherapeutika zu verbessern [33, 43], und wurden mit reduzierter Krebszellproliferation und erhöhter Krebszellapoptose in Verbindung gebracht [21]. Auch bei oraler Mukositis, einer Nebenwirkung der Radio- und Chemotherapie bei Patienten mit Kopf-Hals-Tumoren, stellen Postbiotika eine potenzielle Therapiemöglichkeit dar. Beispielsweise konnten butyrathaltige Spülungen die Schwere der Mukositis verringern und die Heilung beschleunigen. In einige Untersuchungen konnte auch eine Reduktion von Schmerzen und Entzündungssymptomen durch SCFA gezeigt werden [20, 45].

Im Allgemeinen gelten Postbiotika als sicherer, da sie keine lebenden Mikroorganismen enthalten. Jedoch können immunmodulatorische Effekte theoretisch zu unerwünschten Reaktionen führen [31]. Außerdem ist die Wirkung stark von der verwendeten Substanz abhängig und nicht immer vorhersehbar. Obwohl weiterer Forschungsbedarf besteht, deuten diese Ergebnisse darauf hin, dass Postbiotika ergänzend in der Prävention und Therapie verschiedener Erkrankungen eingesetzt werden könnten.

## Fazit für die Praxis

- Das menschliche Mikrobiom, insbesondere das orale Mikrobiom, spielt eine zentrale Rolle für die Mundgesundheit, indem es das Wachstum pathogener Bakterien hemmt und das pH-Gleichgewicht aufrechterhält.
- Eine Dysbiose im oralen Mikrobiom kann Karies, Parodontalerkrankungen und sogar orale Plattenepithelkarzinome begünstigen.
- Präbiotika, Probiotika und Postbiotika bieten vielversprechende Ansätze zur Stabilisierung der oralen Mikrobiota. Sie wirken antimikrobiell gegen pathogene Keime, modulieren das Immunsystem, reduzieren Entzündungen und können ergänzend in der Therapie von Parodontalerkrankungen und Kopf-Hals-Tumoren eingesetzt werden.
- Besonders Postbiotika zeichnen sich durch ihre Stabilität und Sicherheit aus und sind daher vielversprechend für neue Therapieansätze.
- Trotz vielversprechender Ergebnisse ist jedoch weitere Forschung nötig.
